# P53-dependent downregulation of hTERT protein expression and telomerase activity induces senescence in lung cancer cells as a result of pterostilbene treatment

**DOI:** 10.1038/cddis.2017.333

**Published:** 2017-08-10

**Authors:** Rong-Jane Chen, Pei-Hsuan Wu, Chi-Tang Ho, Tzong-Der Way, Min-Hsiung Pan, Hsiu-Min Chen, Yuan-Soon Ho, Ying-Jan Wang

**Affiliations:** 1 Department of Environmental and Occupational Health, College of Medicine, National Cheng Kung University, Tainan, Taiwan; 2 Department of Food Safety/Hygiene and Risk Management, College of Medicine, National Cheng Kung University, Tainan, Taiwan; 3 Department of Food Science, Rutgers University, New Brunswick, NJ, USA; 4 Department of Biological Science and Technology, College of Life Sciences, China Medical University, Taichung, Taiwan; 5 Institute of Food Science and Technology, National Taiwan University, Taipei, Taiwan; 6 Hubei Key Laboratory of Economic Forest Germplasm Improvement and Resources Comprehensive Utilization; Hubei Collaborative Innovation Center for the Characteristic Resources Exploitation of Dabie Mountains; Huanggang Normal University, Huanggang, Hubei, China; 7 Graduate Institute of Medical Sciences, College of Medicine, Taipei Medical University, Taipei, Taiwan; 8 Department of Medical Laboratory Science and Biotechnology, Taipei Medical University, Taipei, Taiwan; 9 Department of Biomedical Informatics, Asia University, Taichung, Taiwan; 10 Department of Medical Research, China Medical University Hospital, China Medical University, Taichung, Taiwan; 11 Graduate Institute of Clinical Medicine, Taipei Medical University, Taipei, Taiwan

## Abstract

Cellular senescence is characterized by permanent cell cycle arrest, triggered by a variety of stresses, such as telomerase inhibition, and it is recognized as a tumor-suppressor mechanism. In recent years, telomerase has become an important therapeutic target in several cancers; inhibition of telomerase can induce senescence via the DNA damage response (DDR). Pterostilbene (PT), a dimethyl ether analog of resveratrol, possesses a variety of biological functions, including anticancer effects; however, the molecular mechanisms underlying these effects are not fully understood. In this study, we investigated the possible mechanisms of PT-induced senescence through telomerase inhibition in human non-small cell lung cancer cells and delineated the role of p53 in senescence. The results indicated that PT-induced senescence is characterized by a flattened morphology, positive staining for senescence-associated-*β* galactosidase activity, and the formation of senescence-associated heterochromatic foci. Telomerase activity and protein expression was significantly decreased in H460 (p53 wild type) cells compared with H1299 (p53 null) cells and p53 knockdown H460 cells (H460-p53-). A more detailed mechanistic study revealed that PT-induced senescence partially occurred via a p53-dependent mechanism, triggering inhibition of telomerase activity and protein expression, and leading to the DDR, S phase arrest and, finally, cellular senescence. This study is the first to explore the novel anticancer mechanism of PT senescence induction via the inhibition of telomerase in lung cancer cells.

Cellular senescence is the specific phenotype in which cells lose the ability to proliferate in response to various mitogens or cellular stresses such as DNA damage, telomere shortening and oxidative stress.^1^ Cells undergoing senescence exhibit characteristics, including irreversible proliferative arrest, resistance to mitogenic and oncogenic stimuli, acquisition of a typical flat and enlarged shape, the increased expression of biomarkers of senescence, such as positive staining of senescence-associated *β*-galactosidase (SA-*β*-gal) activity, accumulation of lysosomes and chromatin remodeling accompanied by the formation of senescence-associated heterochromatin foci (SAHF).^2^ At the molecular level, the senescence response can be triggered by several genetic effectors, converging on the activation of p53 and Rb.^2^ Recent studies indicated that therapy-induced senescence can be achieved at far lower chemotherapeutic doses than those required to induce apoptosis, thus reducing the side effects of anticancer therapy.^3^ Moreover, as cancer cells often develop resistance to apoptosis induced by such therapies, pro-senescence therapy has recently emerged as a novel approach to treat cancers.^4^

Potential intervention targets for the pro-senescence approach are telomerase inhibition, p53 activation, modulation of the cell cycle and the activation of phosphatase and tensin homolog.^5^ Telomerase is an enzyme that is responsible for the maintenance of telomeres, essential structures that cap chromosome ends and protect chromosome stability.^6^ Human telomeres are composed of tandem copies of TTAGGG in DNA repeat sequences and associated proteins, which together form a protective capping complex. Following each cellular division, telomeres become progressively shortened, leading to telomere uncapping triggering the DNA damage response (DDR), which is recognized by the MRE11-RAD50-NBS1 (MRN) complex.^7, 8^ The MRN complex then activates ataxia-telangiectasia mutated (ATM)/ATR and Chk1/Chk2, which in turn phosphorylate and stabilize p53.^4^ Activation of p53 then drives the expression of cyclin-dependent kinase (Cdk) inhibitors, such as p21, which has a direct inhibitory effect on cell cycle progression.^9^ In parallel with p21 expression, other Cdk inhibitors, such as p16, p15 and p27, also induce senescence, as previous described.^10^

Human telomerase is a ribonucleoprotein complex that consists of two essential subunits: the human telomerase reverse transcriptase (hTERT) protein and the small nuclear human telomerase RNA.^6^ The former provides the catalytic activity of telomerase, and the latter provides a template for telomeric repeats.^11^ Although telomerase stabilizes telomeres in human stem cells, cancer cells and reproductive cells, its expression remains in a repressed state in normal human somatic cells.^12^ In addition, telomerase has a pivotal anti-apoptotic role in cancer cells by suppressing apoptotic signaling, thereby circumventing senescence.^12^ Recent studies indicated that telomerase is also expressed in lung cancer and overexpressed in late dysplastic lesions.^13, 14^ Lung cancer is one of the most common cancer types and is responsible for the majority of cancer deaths worldwide. The poor prognosis highlights the urgent need for the development of novel therapeutic strategies for the prevention and treatment of this deadly disease.^3^ Recent studies showed that hTERT polymorphisms are specifically associated with several subtypes of lung cancer.^15^ In addition, strong evidence indicates that hTERT and the epidermal growth factor receptor interact in the etiology of lung cancer.^15^ Consequently, telomerase inhibition-based therapy provides a therapeutic opportunity for lung cancer.^14^

To date, numerous studies focused on either identifying and testing natural agents or synthesizing compounds that inhibit telomerase activity in cancer cells, resulting in the loss of telomere maintenance and induction of senescence.^10^ Pterostilbene (trans-3,5-dimethoxy-4’-hydroxystilbene, PT), a dimethyl ether analog of resveratrol, has similar pharmacologic properties but better pharmacokinetic characteristics (more lipophilic, higher potential for cellular uptake, higher oral absorption and longer half-life) than resveratrol.^16^ The anticancer effects of PT include the inducing of cell cycle arrest, apoptosis, necrosis and autophagy in a few cancer cell lines.^17, 18, 19, 20^ A current molecular docking study performed on PT with the crystal structure of telomerase in cancer cells indicated a good interaction between PT and the active site of telomerase.^21^ However, to the best of our knowledge, no previous study has examined the direct effects of telomerase inhibition via PT treatment in cancer cells. Therefore, it is worthwhile to determine whether low-dose PT suppresses the growth of lung cancer cells via the induction of senescence through inhibition of telomerase activity. In addition, telomere erosion was linked p53 to senescence or apoptosis.^1^ Induction of p53 is pivotal for the initiation and maintenance of senescence mainly through DDR signaling.^1^ Abrogation of DDR or loss of p53 was reported to impair senescence.^1^ Disruption of the normal function of p53 may disrupt the cellular response leading to a reduced therapeutic response or increased overall resistance.^22^ Moreover, p53 mutation was reported significantly correlated with a poor prognosis in lung cancer patients.^22^ Therefore, our current study also investigated the potency of PT-induced senescence by targeting telomerase in lung cancer cells with different p53 phenotypes.

## Results

### PT inhibits cell growth and induces S phase arrest in lung cancer cell lines

**Figure 1:**
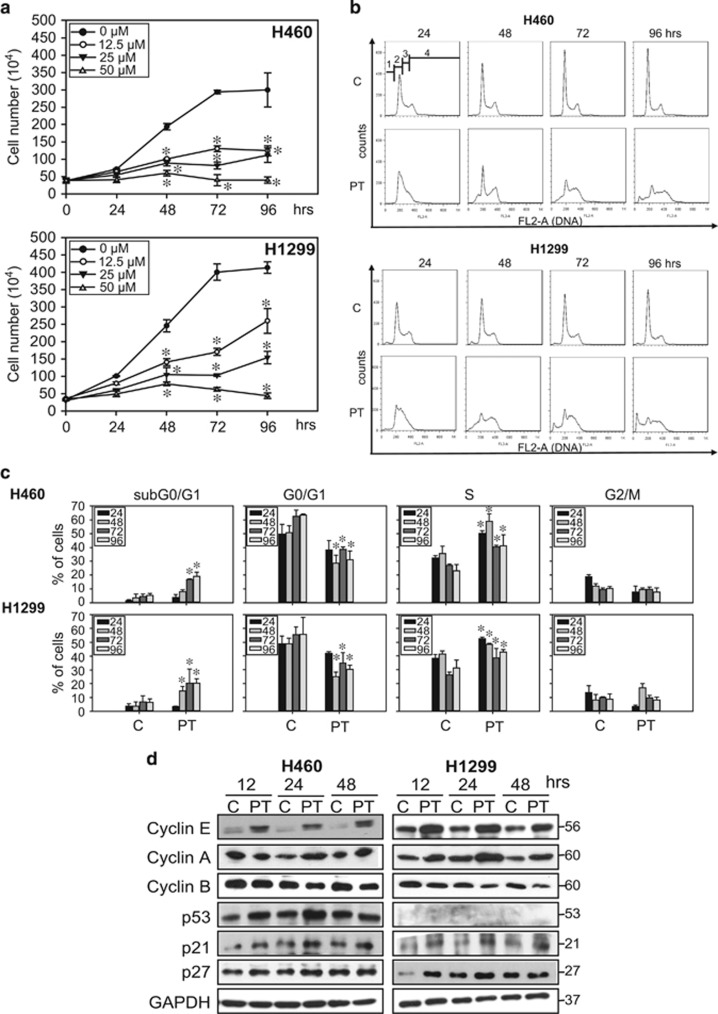
Effects of PT on the growth inhibition and cell cycle arrest in lung cancer cells. (**a**) H460 and H11299 lung cancer cells were plated in six-well plates for 24 h and then treated with different concentrations of PT (0, 12.5, 25 and 50 *μ*M) for 24, 48, 72 and 96 h. Cell numbers were counted daily by Trypan blue exclusion assay. Data represented the mean±S.E.M. of three independent experiments. *P<0.05 compared with the control group (0 *μ*M). (**b**) H460 and H1299 were seeded in six-well plates and treated with 50 *μ*M PT for the indicated times (24, 48, 72 and 96 h). Cells were collected and incubated with 40 *μ*g/ml of PI for 15 min and subjected to flow cytometry analysis to examine the cell distribution at each phase of the cell cycle. Data are presented as representative graphs from three independent experiments. sub-G0/G1, G0/G1, S and G2/M phases are indicated as 1, 2, 3 and 4, respectively. (**c**) The percentage of cells in cell cycle phase was determined using FlowJo 7.6.1 software. Data represent the mean±S.E.M. (*n*=3, *P<0.05 compared with the control (**d**) H460 (left panel) and H1299 cells (right panel) were treated with 50 *μ*M PT for indicated times then cell lysates were isolated and immunoblotted with anti-cyclin E, cyclin A, cyclin B, p53, p21 and p27 antibodies. Membranes were probed with an anti-GAPDH antibody to confirm equal loading of proteins. Representative data from one of three independent experiments are shown

H460 (p53 wild type) and H1299 (p53 null) lung cancer cells were treated with different doses (50, 75 and 100 *μ*M) of PT for 24–96 h. The results indicated that 100 *μ*M PT induced rapid cell death (Supplementary Figures 1A and 1B). PT at a lower dose (50 *μ*M) slowed cell proliferation after 96 h of treatment (Supplementary Figures 1A and 1B), suggesting that a lower concentration of PT is sufficient to inhibit the growth of lung cancer cells. Then, we tested the growth inhibition effects of lower concentrations of PT (12.5, 25 and 50 *μ*M) for 24–96 h. The results showed that lower concentrations of PT significantly decreased cell growth (Figure 1a). Moreover, H460 and H1299 cells became heterogeneous and contained a number of flattened cells with enlarged nuclei compared with normal cells after PT treatment (Supplementary Figure 1C). Accordingly, we hypothesized that a lower concentration of PT (50 *μ*M) may trigger senescence in lung cancer cells. To further elucidate senescence-associated growth inhibition, we analyzed the cell cycle profile of both cells in the presence of PT (50 *μ*M) using fluorescence-activated cell sorting. As shown in Figures 1b and c, lung cancer cells treated with PT showed a slightly increased sub-G0/G1 phase, a decreased G0/G1 phase and gradually accumulated in the S phase over time, compared with mock-treated cells. The results indicated that lung cancer cells entered ‘prolonged arrest’ through the loss of proliferative potential after PT treatment. We then examined the protein expression patterns of cell cycle related regulators in PT-treated cells. Figure 1d shows increased accumulation of cyclin E and cyclin A accompanied by decreases in cyclin B expression, confirming S phase arrest after treatment with 50 *μ*M PT for 12, 24 and 48 h. The repeated experiments showed a subtle increase in expression of cyclin A at 12 h after PT treatment, which was followed by its gradual accumulation in a time-dependent manner (Supplementary Figure 1D). In addition, the expression levels of p-Cdk2 (Tyr15) (an inactivated form of Cdk2) and p53 in H460 cells were also time-dependently upregulated in response to PT treatment (Supplementary Figure 1D). Moreover, the results showed a significant increase of p21 and p27 expression in both cell types (Figure 1d). These data suggest that the p53/p21 axis signaling is likely involved in S phase arrest in PT-treated H460 cells, whereas in H1299 cells, PT-induced p21 expression may be independent of p53 activation (Figure 1d).

### p53 wild-type H460 cells are more sensitive to PT-induced senescence than p53 null H1299 cells

**Figure 2:**
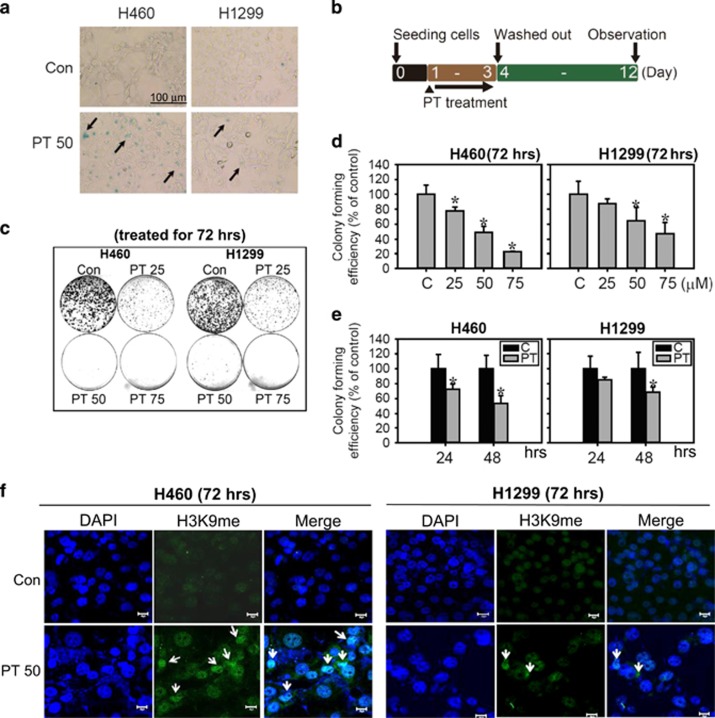
PT-induced senescence in lung cancer cells. Senescence morphology of H460 and H1299 cells treated with 50 *μ*M PT for 48 h. Cells were stained for *β*-gal. The representative images are shown with arrows indicating senescent morphology (**a**), bar: 100 *μ*m. The experiment scheme for measuring loss of replication and regenerative potential (RP) was presented in (**b**). Cells were seeding overnight and then treated with or without 50 *μ*M PT for 72 h. PT was then removed and cells could recover for additional 9 days (total period of 12 days). Then, colonies were stained with crystal violet (**c**). (**d**) Lung cancer cells were treated with 25, 50 or 75 *μ*M PT for 72 h then PT was removed and the cells were allowed to recover for additional 9 days. (**e**) The colony-forming efficiency was also examined in lung cancer cells treated with 50 *μ*M PT for 24 and 48 h and then cultivated in drug-free medium for additional 9 days. Data represent the mean±S.E.M. (*n*=3, *P<0.05 compared with the control). *Y* axis represents % decreases in the number of colonies relative to control. (**f**) Immunofluorescence analysis of the senescent heterochromatin foci stained with H3K9me3 (green) and with DAPI (blue) to visualize DNA in H460 and H1299 cells treated with PT (50 *μ*M) for 72 h (Bar: 20 *μ*m)

Based on the proliferation arrest and morphological changes, we propose that the lung cancer cells underwent senescence in response to PT. We then assayed several markers for senescence. The first marker was SA-*β*-gal activity, which is the most ubiquitous cellular senescence marker.^23^ The enlarged cells exhibited an increase in SA-*β*-gal activity (Figure 2a), with up to 40% of H460 cells staining positive, compared with 20% of H1299 cells under the same treatment for 48 h (Supplementary Figures 2A and 2B). Then, we investigated the replicative and regenerative potential in PT-treated cells using a colony formation assay because colony formation ability is lost in senescent cells even in the presence of a mitogen.^24^ Lung cancer cells were treated with PT for 72 h, and PT was washed out with fresh medium (Figure 2b). Indeed, the results showed a marked retardation of replicative potential after an additional 9-day incubation in lung cancer cells in the absence of PT (Figure 2c). PT significantly suppressed colony-forming activity in a dose- and time-dependent manner compared with the different p53 status of lung cancer cells, such that, H1299 cells displayed higher colony-forming efficacy than H460 cells under the same PT treatment conditions (Figures 2d and e). Another hallmark of senescence is the formation of SAHF, which is known as areas of condensed and transcriptionally silenced DNA that can be detected by co-staining of DAPI and tri-methylation of histone H3 on lysine 9 (H3K9me3).^25^ Figure 2f shows the positive staining for H3K9me3 cells is also significantly increased in H460 cells compared with a slight increase in H1299 cells under the same treatment conditions. These results clearly showed that a lower concentration of PT-induced cellular senescence in lung cancer cells and that p53 wild-type H460 cells were more sensitive to PT-induced senescence than p53 null H1299 cells.

To further confirm the pro-senescence potency of PT, A549 lung cancer cells and MCF7 breast cancer cells were treated with 50 *μ*M PT for 24 h and the SA-*β*-gal activity was quantified by C_12_FDG staining (Supplementary Figures 2C and 2D). The results showed that PT induced approximately 35% senescence in both cells, which clearly demonstrates that PT is a promising senescence-promoting agent in a variety of cancer cell lines.

### p53 contributes to PT-mediated telomerase inhibition and senescence induction

**Figure 3:**
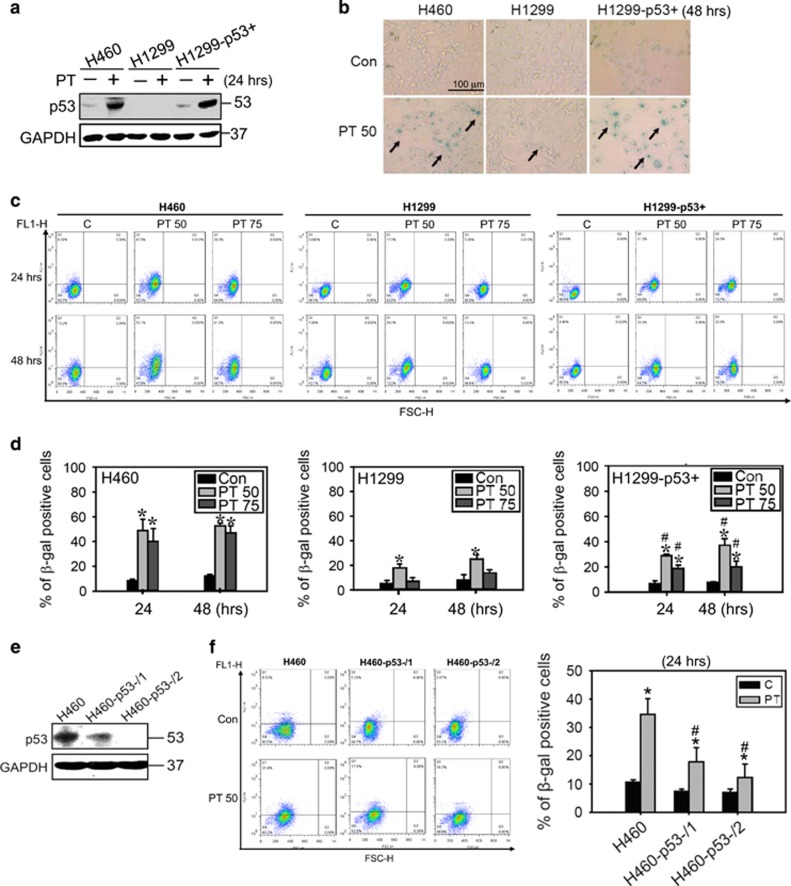
PT-induced senescence in lung cancer cells mediated by p53. (**a**) Protein expression of p53 in H460, H1299 and H1299-p53+ lung cancer cells after treated with 50 *μ*M PT for 24 h. (**b**) Senescence morphology of H460 and H1299 cells treated with 50 *μ*M PT for 48 h. Bar: 100 *μ*m. (**c**) H460, H1299, and H1299-p53+ cells treated with 50 and 75 *μ*M PT for 24 and 48 h and then incubated with C_12_FDG to detect SA-*β* gal activities by flow cytometry. *X* axis: FSC-H, *Y* axis: FL1-H. (**d**) The percentage of SA-*β* gal-positive cells detected by C_12_FDG staining is shown. Data represent the mean±S.E.M. of three independent experiments. *P<0.05, significantly higher than control group in different time course category. ^#^*P*<0.05, significantly higher than H1299 groups. (**e**) Western blot analysis showed the expression of p53 in H460 cells and p53 stable knockdown cell lines (H460-p53-/1 and H460-p53-/2). (**f**) The percentage of SA-*β* gal-positive cells detected by C_12_FDG staining was shown in H460, H460-p53-/1 and H460-p53-/2 cells treated with 50 *μ*M PT for 24 h. Data represent the mean±S.E.M. of three independent experiments. *P<0.05, significantly higher than control groups, ^#^*P*<0.05, significantly lower than H460 PT-treated groups. *X* axis: FSC-H, *Y* axis: FL1-H

We therefore examined whether the p53 status in lung cancer cells can affect senescence induction by PT. To this end, H1299 cells with stable ectopic expression of p53 (H1299-p53+) were used. After treatment with PT for 24 h, the expression of p53 was significantly increased in H460 and H1299-p53+ cells (Figure 3a). As expected, PT induced more senescent characteristics in H1299-p53+ cells than in p53 null H1299 cells, determined by SA-*β*-gal activity (Figure 3b and Supplementary Figure 3) and C_12_FDG staining (Figure 3c). Quantification of C_12_FDG staining showed that 50 *μ*M PT induced 30% and 40% senescence at 24 and 48 h, respectively, in H1299-p53+ cells, whereas no >20% senescence was observed in H1299 cells (Figure 3d). In addition, we established stable p53 knocked down H460 cell lines (H460-p53-/1 and H460-p53-/2) using two different p53 short hairpin RNA (shRNA) constructs (Figure 3e). As shown in Figure 3f, knockdown of p53 resulted in a significant decrease in SA-*β*-gal activity by a maximum of approximately 20% in H460-p53-/2 cells after treatment with 50 *μ*M PT for 24 h. The results showed that the p53-mediated pathway enhances PT-induced senescence.

**Figure 4:**
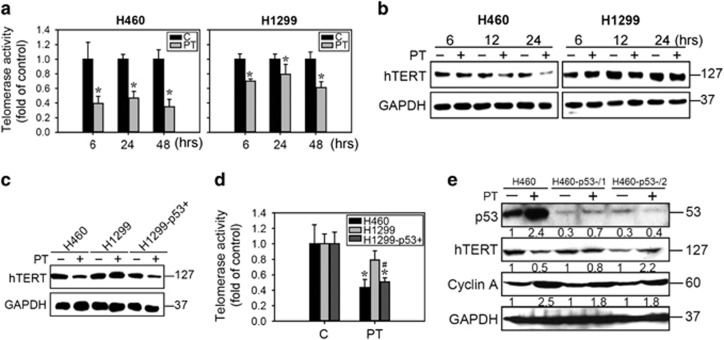
PT inhibited telomerase enzyme activity and protein expression in lung cancer cells. (**a**) H460 and H1299 cells were treated with 50 *μ*M PT for the indicated times (6, 24 and 48 h) and telomerase enzyme activity were measured using a TRAPeze RT Telomerase Detection Kit. (**b**) Cell lysates extracted from H460 and H1299 cells treated with 50 *μ*M PT for 6, 24 and 48 h were submitted to western blot analysis to detect the protein expression of hTERT. Equal loading was confirmed by GAPDH staining. (**c**) Western blot analysis for hTERT expression and (**d**) telomerase enzyme activity was measured by TRAPeze RT Telomerase Detection Kit in H460, H1299 and H1299-p53+ cells treated with 50 *μ*M PT for 24 h. Data represented the mean±S.E.M. of three independent experiments. *P<0.05, compared with control groups. ^#^*P*<0.05, significantly lower than H1299 groups. (**e**) Protein expression in H460, H460-p53-/1 and H460-p530-/2 cells treated with 50 *μ*M PT for 24 h. The membrane was probed with anti-GAPDH to confirm equal loading of proteins. The number below each line indicates the relative intensity of protein expression compared with H460 control groups (p53) or each group without PT treatment (hTERT and cyclin A) (defined as 1)

As the S phase is tightly regulated to ensure genome duplication and stability, alteration of the replication process by replicative stress may induce S phase checkpoint activation. Replicative stress induced by telomerase inactivation was implicated in the onset of cellular senescence.^26^ We next examined the telomerase inhibitory effects of PT in H460 and H1299 cells. As shown in Figure 4, following PT treatment for 6–48 h, both hTERT activity and protein expression in H460 cells were significantly decreased compared with H1299 cells (Figures 4a and b). We further confirmed whether the inhibition of hTERT activity and expression is mediated by p53, and the results revealed that hTERT expression and activity were reduced in H1299-p53+ cells similar to H460 cells treated with PT (Figures 4c and d). Next, we analyzed hTERT and cyclin A expression in H460, H460-p53-/1 and H460-p53-/2 cells. We observed that the expression of hTERT was decreased in H460 cells treated with PT, whereas the expression of hTERT was increased in p53 knockdown cells after PT treatment compared with H460 PT-treated groups (Figure 4e). Importantly, p53 knockdown reduced cyclin A accumulation after PT treatment. These results provide evidence that supports the requirement of p53 for hTERT inhibition and, may explain the mechanism underlying PT-induced senescence.

### hTERT overexpression confers resistance to senescence induced by PT

**Figure 5:**
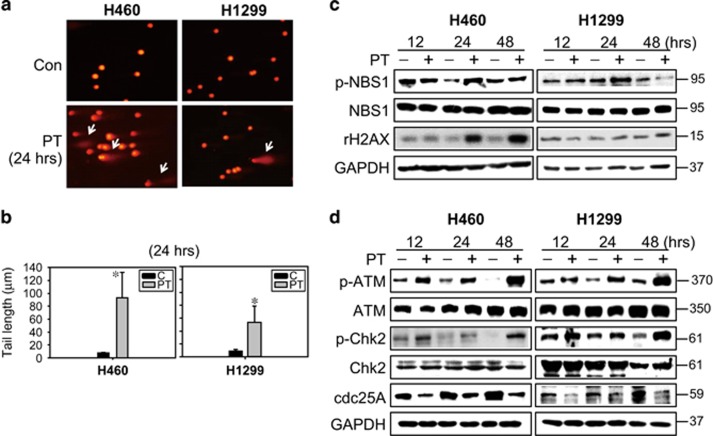
PT-induced DNA damage in lung cancer cells. (**a**) Photomicrography of H460 and H1299 cells treated with 50 *μ*M PT for 24 h and analyzed using comet assay. (**b**) DNA damage level expressed as tail length (*μ*m) calculated using Komet 5.5 software (Kinetic Imaging Ltd., London, UK) after 24 h of 50 *μ*M PT treatment in H460 and H1299 cells (mean±S.E.M., *n*=3, *P<0.05 compared with control groups). (**c**) H460 and H1299 cells were treated with 50 *μ*M PT for 12, 24 and 48 h. Total-cell lysates were subjected to western blot analysis for (**c**) DNA damage proteins and (**d**) DNA sensor kinase (ATM), cell cycle checkpoint kinase (Chk2), and cell cycle regulatory protein (cdc25A). Membranes were probed with an anti-GAPDH antibody to confirm equal loading of proteins. Results are representative of three independent experiments

The inhibition of telomerase can lead to induction of the DDR, replication fork stalling, activation of the intra-S-phase checkpoint and senescence.^25^ As shown in Figures 5a and b, we provide the evidence of DNA damage in cells following hTERT inhibition using a comet assay. A marked increased in DNA strand breaks (DSBs) in PT-treated H460 cells compared with H1299 cells under the same treatment conditions was observed. Other marker for DSBs including activation of DNA damage checkpoint factor NBS1 and γH2AX (the phosphorylated H2AX) were detected (Figure 5c). In addition, DDR activated ATM and its downstream kinase Chk2, and the subsequent inactivation of cdc25A, which is Chk2 substrate^27^ (Figure 5d).

**Figure 6:**
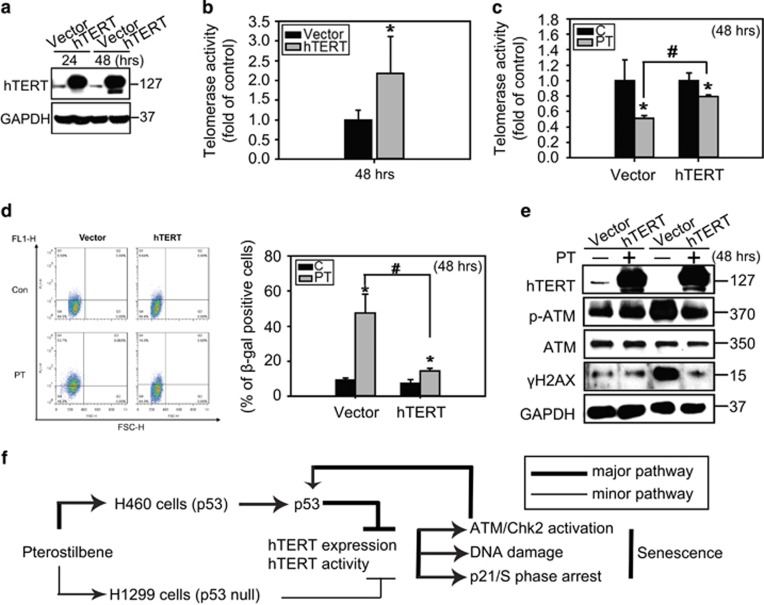
Exogenous telomerase expression rescued telomerase activity and decreased senescence induced by PT. (**a**) H460 cells were transiently transfected with either pcDNA-3.1 vector or pcDNA-3.1 hTERT-3HA plasmid for 24 and 48 h. Cell lysates were analyzed for the expression of hTERT by western blot analysis and (**b**) telomerase enzyme activity. (**c**) The telomerase enzyme activity of the vector or hTERT transfected H460 cells treated with 50 *μ*M PT for 24 h were measured using a TRAPeze RT Telomerase Detection Kit. (mean±S.E.M., *n*=3, *P<0.05, significantly lower than control groups. ^#^*P*<0.05, significantly higher than vector groups). (**d**) The SA *β*-gal activity of cells treated as in (**b**) was stained with C_12_FDG and analyzed by flow cytometry. *X* axis: FSC-H, Y axis: FL1-H. Data represented the mean±S.E.M. of three independent experiments. *P<0.05, compared with control groups. ^#^*P*<0.05, significantly lower than vector groups. (**e**) Cells were treated as described in (**b**) and immunoblotting was performed with anti-hTERT, p-ATM, ATM, γH2AX, and GAPDH antibodies. Results are representative of three independent experiments. (**f**) Proposed model summarizing PT-induced senescence in lung cancer cells. In p53 wild-type H460 cells, PT inhibits hTERT enzyme activity and protein expression resulting in the subsequent induction of DNA damage, activation of ATM/Chk2 and p53, and S phase arrest. Activation of p53 positive feedback provokes hTERT downregulation, resulting in senescence in H460 cells. Interestingly, PT slightly inhibited hTERT enzyme activity resulting in less senescence in H1299 cells, suggesting that PT-induced senescence in lung cancer cells partially through p53-mediated hTERT inhibition

To gain further insight into whether hTERT inhibition contribute to PT-induced senescence, we established an hTERT-overexpressing H460 cell line (Figure 6a). hTERT protein expression and activity were significantly increased in H460-hTERT+ cells compared with the H460 vector control cells (Figures 6a and b). Enforcement of hTERT expression rescued telomerase activity (Figure 6c) and prevented PT-induced senescence as determined by C_12_FDG staining and SA-*β*-gal staining compared with H460 vector control cells (Figure 6d). In addition, we found that the activation of ATM and induction of γH2AX were decreased in H460-hTERT+ cells after PT treatment (Figure 6e), indicating that hTERT overexpression rescues PT-induced DNA damage and senescence. These findings highlight the impact of telomerase inhibition by PT, which may initiate senescence by enforcing permanent cell cycle arrest when the ATM/Chk2 DNA damage checkpoint pathway is activated.

## Discussion

Cellular senescence inhibits tumor progression *in vivo*, thus making it an attractive therapeutic target for cancer.^10^ The drug concentration of anticancer agents or the dose of radiation required to induce senescence is lower than that necessary to kill cells; therefore, senescence-inducing treatments provide the advantage of enhancing treatment efficacy and reducing side effects in anticancer therapy.^9^ In this study, we demonstrated a novel anticancer effect of PT through senescence induction, which was preferentially observed in p53+ lung cancer cells. The possible underlying mechanism is as follows: PT→inhibition of telomerase activity and protein expression→DNA damage→ATM/Chk2/p53 activation→p21 induction→prolonged S phase arrest→senescence (Figure 6f). In addition, ATM/Chk2-activated p53 may act as a feedback regulation to inhibit hTERT expression to increase DNA damage and senescence in H460 cells (Figure 6f). To the best of our knowledge, this is the first report investigating the induction of senescence in lung cancer cells by primarily targeting hTERT using low-dose PT.

With regard to the role of hTERT in lung cancer biology, ectopic expression of hTERT in primary lung epithelial cells immortalizes cells, indicating that increased hTERT activity may increase the cell proliferation capacity of normal cells.^15^ Multiple studies verified the hTERT expression level using qRT-PCR and showed that hTERT expression is significantly higher in tumor tissues of non-small cell lung cancer than in normal tissues.^15^ Mechanistic studies indicated that hTERT promotes epithelial proliferation through transcriptional pathways, including the Myc and Wnt pathways.^15^ Therefore, downregulation of hTERT subsequently reduced telomerase activity and led to lung adenocarcinoma apoptosis and reduced tumor size, indicating that telomerase is an attractive target for lung cancer therapy.^28^ Therapeutic inhibition of telomerase can be achieved by a variety of mechanisms, including direct inhibition, a vaccine-generated immune response, and induction of the DNA response by T-oligos.^29^ Our current study provides additional evidence to indicate that PT inhibits telomerase expression and activity, suggesting that PT could be useful clinically.

After telomerase inhibition, DDR was increased in H460 cells (Figures 5a and c), and led to activation of the ATM and Chk2 pathways (Figure 5d). DNA damage is a primary inducer of replication fork stalling that leads to activation of the intra-S-phase checkpoint through ATM-Chk2-cdc25A-Cdk pathways.^25^ We suggest that a low concentration of PT-induced S phase arrest in lung cancer cells may be related to telomerase inhibition (Figure 1). Indeed, the expression of cyclin A was gradually increased in a time-dependent manner at 12, 24 and 48 h after PT treatment (Supplementary Figure 1D). According to a previous study indicating that the expression of cyclins is a sequential and periodic event,^30^ we show that PT treatment slightly increased the expression of cyclin A at 12 h post-treatment, which continue to accumulate until 48 h post-treatment. Furthermore, S phase arrest may occur because of phosphorylation of Cdk2 at Tyr15, which inhibits activity of the cyclin A/Cdk2 complex.^30^ In this regard, activation of cyclin A/Cdk2 has an important role in driving the progression of S phase. Our results showed a time-dependent accumulation of cyclin A and inactivation of Cdk2, eventually causing cell cycle arrest. Taken together, our study is the first to delineate possible mechanisms of S phase arrest by low-dose PT treatment, and suggests that S phase arrest may be a permanent arrest characteristic of senescence. Moreover, ATM activates and stabilizes p53 via a complex signaling pathway, such as Chk2 activation or MDM2 degradation.^8^ p53 activation further drives the expression of proteins involved in the cell cycle checkpoint, such as p21.^8^ Consistence with these studies, we showed that PT-induced S phase arrest was likely because of degradation of cdc25A and p53-p21 activation following activation of ATM/Chk2 (Figures 1 and 5d).

One of the most well-established p53-target genes in regulating senescence is p21, which is an inhibitor of cell cycle progression.^1^ However, the molecular mechanisms of p53-mediated senescence remain elusive.^1^ In addition to p21 induction, we found that p53 acted as an upstream negative regulator of hTERT activity and expression in H460 (p53 wild type) but not H1299 (p53 null) lung cancer cells (Figure 4). Consistent with our results, it was previously shown that p53 is essential for hTERT promoter repression, in which it binds to Sp1 to render it inaccessible to hTERT activation.^31^ Lack of p53 reduced senescence and partially abrogated the inhibition of hTERT expression and activity (Figure 4e). Nevertheless, re-expression of p53 in H1299 cells potently inhibited hTERT expression and activity (Figures 4c and d). These findings emphasize that p53 regulates a plethora of target genes affecting several pathways in PT-treated senescent lung cancer cells that are likely to be highly involved in the regulation of hTERT protein expression and activation. However, we also showed that PT slightly inhibited hTERT activity and expression in p53 null H1299 cells (Figure 4). Consistent with our results, it was reported that resveratrol downregulates telomerase activity and nuclear levels of hTERT in MCF7 cells via suppression of c-Myc in a p53-independent pathway.^32^ Xu *et al.*^31^ showed that the undirected mechanism of transcriptional repression of hTERT could occur via the p21/Rb/E2F axis and the recruitment of histone deacetylase-containing pocket protein complexes. Other transcription factors trigger feedback regulation of hTERT expression including pRb/E2F, Wnt/*β*-catenine, NF*κ*B and PI3K/Akt molecules.^33^ In addition, hTERT activation pathways could also be influenced by Cdk2, Cdk4 and AKT.^6^ These studies support our findings that PT-induced senescence, by targeting of hTERT, may be mediated by both p53-dependent and -independent repression mechanisms. As hTERT is an important target for the senescence-inducing effect of PT, it is worth conducting further studies to investigate the regulatory mechanisms of hTERT by PT via a p53-independent pathway.

Interestingly, induction of p21 and p27 by PT was also found and may be associated with senescence in H1299 cells, indicating p53-independent upregulation of p21 and p27 (Figure 1). p21 is primarily involved in cell cycle arrest via inhibition of Cdk activity, and can be regulated by p53-independent mechanisms, leading to S phase arrest and downregulation of cdc25A.^34^ It was also reported that p21 can inhibit Cdk, which in turn leads to phosphorylation of Rb and consequent inhibition of E2F, resulting in p53-independent senescence.^26^ Moreover, p27 inhibits Cdk activity during senescence.^35^ Consistent with our results, increased expression of p21 was also observed in H1299 cells via various mechanisms.^36, 37, 38, 39, 40^ An *et al.*^36^ indicated that forkhead box A1/2 (FOXA1/2) is a crucial transcription factor that activates p21 expression by directly binding to the p21 promoter in p53 null H1299 cells. Another study indicated that hRAD9 is a potential tumor suppressor in breast and lung cancers, which could upregulate p21 expression independent of p53.^39^ In addition, other factors, such as miR-512-5p,^37^ miR-132/212,^38^ and N-myc downstream regulated gene 1,^41^ were reported to control p21 expression in a p53-independent manner. Although the precise mechanisms by which PT induces p21 and p27 expression through a p53-independent pathway remains unclear, we propose that the induction of p21 and p27 occurs through a p53-independent pathway that drives senescence in p53 null H1299 cells (Figure 1).

Our previous study indicated that PT inhibits the growth of cancer cells by inducing apoptosis and autophagy at higher doses.^17^ However, the challenge for an apoptosis-inducing strategy is that the concentration required for apoptosis in tumor cells *in vitro* is not attainable *in vivo*. Senescence can be achieved at far lower doses for anticancer agents than those required to induce apoptosis, thereby, reducing the side effects of anticancer therapy. In this study, we showed a novel antitumor effect of a lower concentration of PT via downregulated hTERT expression and hTERT activity leading to senescence. PT-induced senescence usually relies on the activation of p53, but PT can also induce senescence in p53 null cells, although the relative mechanisms remain unclear. This could open a door to the treatment of chemoresistance tumors that carry mutations in the p53 pathway. In a similar manner, it was reported that a variety of phytochemicals, including curcumin, genistein and fisetin, inhibit the expression of telomerase components or induce senescence in a variety of cancer cells.^6^ Phytochemicals are attractive anticancer agents because of their accessibility in diets, low cost and low toxicity.^6^ Therefore, we suggest an alternative strategy for the use of PT, which is found in the diet, to treat lung cancer through the induction of senescence via the downregulation of hTERT expression and activation, and the relative mechanisms by which this occurs warrant further investigation.

## Materials and methods

### Cell culture

The human lung cancer cell line H460 (p53 wild type, ATCC HTB177), H1299 (p53 null, ATCC CRL-5803) and H1299 cells transfected with p53 stable clones (H1299-p53+) were kindly provided by Professor Jiunn-Liang Ko (Institute of Medical and Molecular Toxicology, Chung Shan Medical University, Taichung, Taiwan). hTERT-overexpressed H460 cells were transient transfected with pcDNA-3xHA-hTERT (Plasmid #51637, Addgene, Cambridge, MA, USA) and the control cells were transient transfected with pcDNA-3.1 Vector (Invitrogen, Thermo Fisher Scientific Inc., Waltham, MA, USA) as vector control. The p53 knockdown shRNAs were obtained from the National RNA interference Core Facility located at the Institute of Molecular Biology/Genomic Research Centre, Academia Sinica (Nankang, Taipei, Taiwan). The human library is referred to as TRC-Hs 1.0. Individual clones were identified as shRNA TRCN0000003753 and TRCN0000003756. All cell lines were maintained in 10 cm^2^ dishes in RPMI (Sigma-Aldrich, St. Louis, MO, USA) supplemented with 100 U/ml penicillin, 100 *μ*g/ml streptomycin (Life Technologies, Inc., Gaithersburg, MD, USA), 10% heat-inactivated fetal calf serum (HyClone, South Logan, UT, USA), sodium pyruvate and glutamine (Life Technologies, Inc.). PT was provided by Dr. Chi-Tang Ho (Department of Food Science, Rutgers University, New Brunswick, NJ, USA) and the purity of PT is 96%.

### Cell cycle analysis

After treatment, cells were washed with PBS, resuspended by trypsinization and fixed with 75% ethanol for at least 4 h. After fixation, the cells were washed with PBS and stained with 40 *μ*g/ml propidium iodide (PI) for 30 min. PI immunofluorescence was measured by flow cytometry (FACScan, Becton Dickinson, San Jose, CA, USA) and cell cycle distribution was quantified using FlowJo 7.6.1. software (Tree Star Inc, Ashland, OR, USA). As shown in Figure 1b, cell population percentages were analyzed by the DNA content, in which sub-G0/G1, G0/G1, S and G2/M phases are indicated as 1, 2, 3 and 4, respectively.

### Cytochemical staining for SA-*β* gal activity

The senescent cells expressed beta-galactosidase activity that was detectable at pH 6.0 and is now called ‘senescence-associated-*β* galactosidase’ activity (SA-*β* gal).^23^ After PT treatment, the cells were washed with PBS, and fixed with fixation solution (2% formadehyde and 0.2% glutaraldehyde in PBS buffer). The fixation buffer was then removed and the cells were incubated with staining solution (containing 40 mM citric acid/Na phosphate buffer, 5 mM K_4_[Fe(CN)_6_]3H_2_O, 5 mM K3[Fe(CN_6_)], 150 mM sodium chloride, 2 mM magnesium chloride and 1 mg/ml X-gal) at 37 °C without CO_2_ for 12 h. After incubation, the cells were washed with PBS and once with methanol and the dish was air dried. The percentage of SA-*β* gal-positive cells was determined by counting the number of blue cells in the dishes under phase contrast microscopy at 200x magnification and the representative fields were photographed.

### Fluorescence detection of SA-*β* activity by C_12_FDG staining

Senescent cells can be detected in living cells, and quantified by flow cytometry using the fluorogenic substrate C_12_FDG to increase the assay sensitivity.^23^ In this procedure, the internal pH of lysosomes is increased to ~pH 6 using the lysosome inhibitor chloroquine. Before cells were harvested, cells were treated with 0.3 M chloroquine for 2 h, and then C_12_FDG (33 *μ*M) was added to the medium and incubated for 2.5 h. The cells were then washed, resuspended by trypsinization and analyzed by flow cytometry (FACScan, Becton Dickinson). The C_12_FDG signal was measured using FL1 detector and quantified with FlowJo 7.6.1 software.

### Colony formation assay

Two thousand cells were plated in 10 cm^2^ dishes. The next day, cells were treated with different concentrations of PT for the indicated times (24, 48 or 72 h). After treatment, the medium was removed. The cells were washed and grown in the fresh medium for an additional 9 days to allow the formation of cell colonies. The colonies were then fixed and stained with 0.5% crystal violet in methanol for 30 min. The plates were photographed, and the number of colonies (≥50 cells) was scored under microscopy.^42^ The number of colonies was then extrapolated to the area of the entire dish.

### Comet assay

The comet assay is single-cell electrophoresis to detect DNA damage.^43^ Briefly, cells were harvested and suspended in 1% low-melting point agarose at the density of 1 × 10^6^ cells/ml. The cell suspension was then pipetted onto slides pre-coated with 0.5% normal melting point agarose. The third layer of agarose was added to the cell containing slides. Then slides were immersed in cold lysis buffer for 30 min at 4 °C and subjected to electrophoresis for 15 min.^43^ The slides were then washed, stained with ethidium bromide (20 *μ*g/ml) and scored under a fluorescence microscope at 100 ×, with an excitation filter of 515–560 nm and a barrier filter of 590 nm. DNA damage levels of 100 randomly selected cells were scored for tail features as described in the reference Wang *et al.*^43^

### Immunofluorescence staining of H3K9me3

Cells were plated in a six-well plate at a density of 3 × 10^4^ per well overnight. After treatment with PT for the indicated times, cells were fixed in 4% formaldehyde for 20 min and then washed with PBS. Cells were then incubated with blocking solution (10% fetal calf serum) for 30 min, with the primary H3K9me3 antibody for 1 h, with fluorophore-conjugated secondary antibody for 1 h, mounted with Vectashield Mounting Medium with DAPI (Vector Laboratories, # H-1600, Peterborough, UK), and then analyzed using a fluorescence microscope.

### Western blot analysis

Whole-protein extracts were separated on 6–15% SDS-polyacrylamide gels and transferred to polyvinylidene difluoride membranes (Merck Millipore, Darmstadt, Germany). Membranes were probed with primary antibodies, washed with 1 × TBST, and then probed with horseradish peroxidase-conjugated anti-mouse or anti-rabbit secondary antibodies. Immunoreactive proteins were visualized with the enhanced chemiluminescence detection system (PerkinElmer Life Science, Inc., Waltham, MA, USA) and BioMax LightFilm (Eastman Kodak Company, New Heaven, CT, USA), according to the manufacturer’s instructions.

### Reverse transcription (RT)-PCR and telomerase activity assays

Cells were plated in a six-well plate at a density of 1 × 10^5^ per well overnight and then treated with PT 50 *μ*M for 6, 24 and 48 h. Telomerase enzyme activity was measured using TRAPeze RT Telomerase Detection Kit (Merck Millipore, cat no. S7710) following the manufacturer’s protocol. Briefly, 0.75 *μ*g protein from each cell extraction was mixed with the TRAP PCR mixture and the PCR was performed using the StepOnePlus Real-Time PCR System (Thermo Fisher Scientific Inc.). The values for hTERT activity were normalized to the GAPDH housekeeping control and the activity of control groups was determined as 100%.

### Statistical analyses

Results are expressed as the mean±S.E.M. Experimental data were analyzed using Student’s *t*-test. Differences were statistically significant when the *P*-value was <0.05.
