# Supplementary figures and images for: P53-dependent downregulation of hTERT protein expression and telomerase activity induces senescence in lung cancer cells as a result of pterostilbene treatment
Source: Cell Death Dis. 2017 Aug 10;8(8):e2985–. doi: 10.1038/cddis.2017.333 (PMC5596539; doi:10.1038/cddis.2017.333)

Supplementary Data Figure 1

(A)

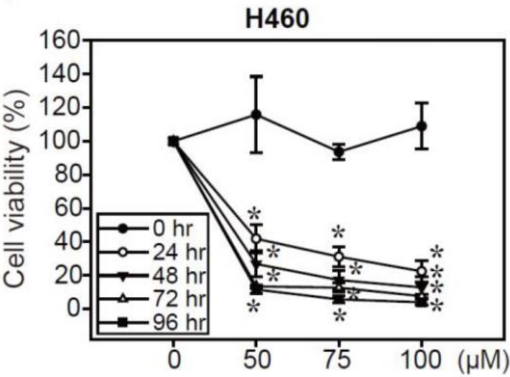

(B)

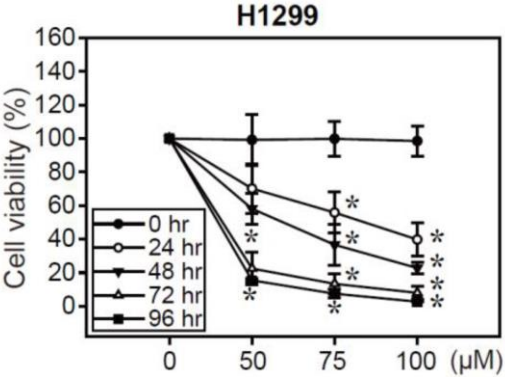

(C)

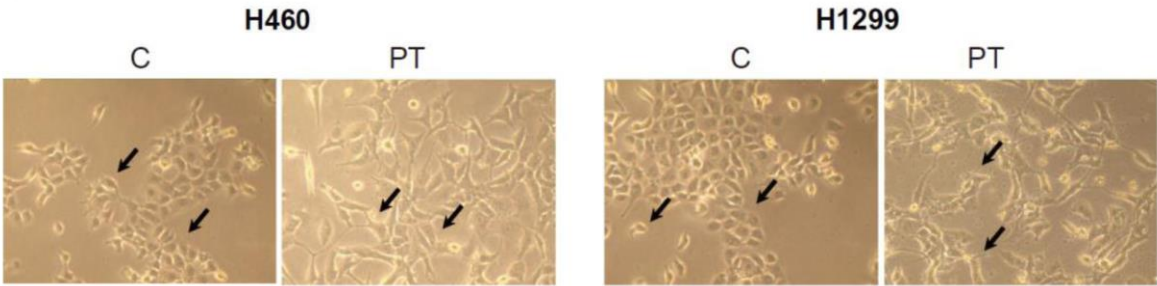

(D)

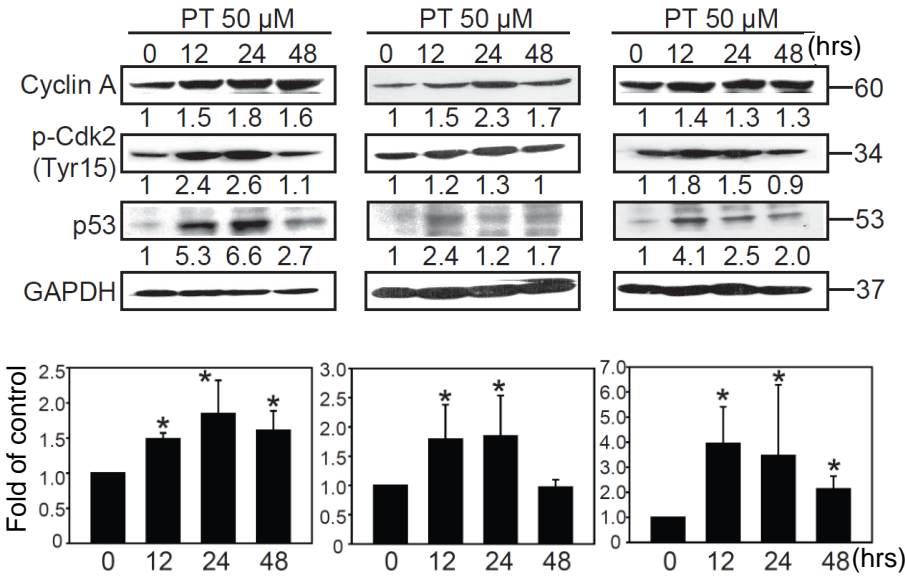

Supplement: Supplementary data Figure 1 [file cddis2017333x1.pdf]

Supplementary Data Figure 2

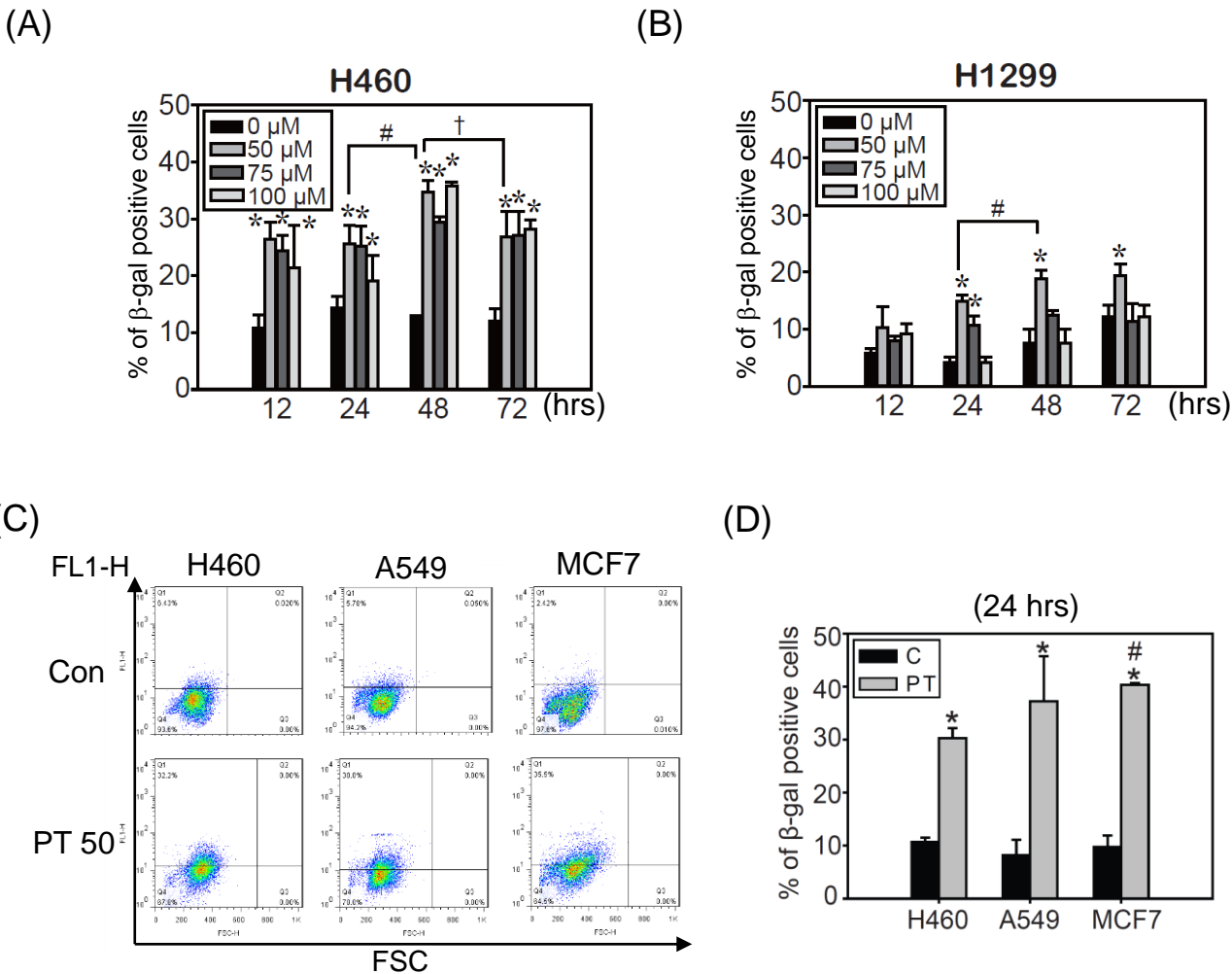

Supplement: Supplementary data Figure 2 [file cddis2017333x2.pdf]

Supplementary data Figure 3

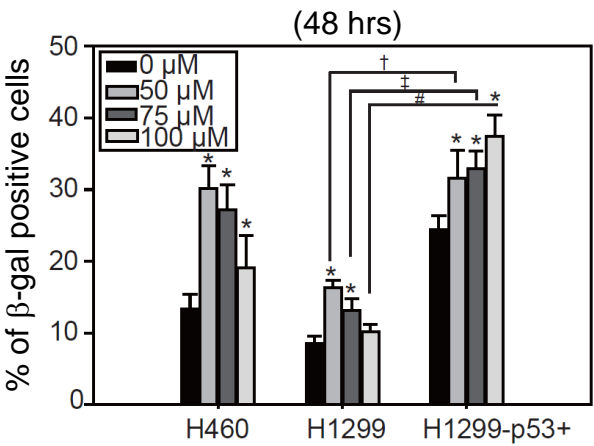

Supplement: Supplementary data Figure 3 [file cddis2017333x3.pdf]
