# Supplementary material for: P53-dependent downregulation of hTERT protein expression and telomerase activity induces senescence in lung cancer cells as a result of pterostilbene treatment
Source: Cell Death Dis. 2017 Aug 10;8(8):e2985–. doi: 10.1038/cddis.2017.333 (PMC5596539; doi:10.1038/cddis.2017.333)
Supplement: Supplementary Figure legend [file cddis2017333x4.docx]

**Supporting Data Figure 1:** Cell viability was reduced in (A) H460 and (B) H1299 lung cancer cells after treatment with different concentrations (50, 75, 100 μM) of PT for 24, 48, 72 and 96 hrs as examined by trypan blue exclusion assay. Data represented the mean ± SEM of three independent experiments. **p*<0.05 compared to the control group (0 μM). (C) Morphologic changes of H460 and H1299 cells treated with 50 μM PT for 48 hrs. Photographs were taken under 40x magnifications. The arrows represent the flattened cells after PT treatment. (D) Three independent results of Cyclin A, p-Cdk2 (Tyr15), and p53 expression in H460 treated with 50 μM PT for 0, 12, 24, and 48 hrs. The number below each line indicated the relative intensity of protein expression compared with the 0 hr control groups. The membrane was probed with anti-GAPDH to conﬁrm equal loading of proteins. The statistical data were shown below. **p*<0.05 compared to the control groups (0 hr).

**Supporting Data Figure 2:** The percentage of SA-β gal positive staining cells in (A) H460 and (B) H1299 cells treated with PT (50, 75, 100 μM) for 12, 24, 48, and 72 hrs. Data represented the mean ± SEM of three independent experiments. **p*<0.05 compared to the control group (0 μM). ^#^*p*<0.05, significantly higher than in groups treated with PT for 24 hrs. †*p*<0.05, significantly higher than in groups treated with PT for 72 hrs. (C) The SA β-gal activity of H460, A549 and MCF7 cells treated with 50 μM PT for 24 hrs was stained with C_12_FDG and analyzed by flow cytometry. X axis: FSC-H, Y axis: FL1-H. (D) Data represented the mean ± SD of three independent experiments. **p*<0.05, compared with H460 control groups. ^#^*p*<0.05, significantly higher than H460 PT treated groups.

**Supporting Data Figure 3:** The percentage of SA-β gal positive staining cells in H460, H1299, and H1299-p53+ cells treated with PT (50, 75, 100 μM) for 48 hrs. Data represented the mean ± SEM of three independent experiments. **p*<0.05 compared to the control group (0 μM). †*p*<0.05 significantly higher than H1299 PT 50 μM treated groups. ‡*p*<0.05 significantly higher than H1299 PT 75 μM treated groups. ^#^*p*<0.05, significantly higher than PT 100 μM treated groups.
